# Personality traits and academic performance: Correcting self-assessed traits with vignettes

**DOI:** 10.1371/journal.pone.0248629

**Published:** 2021-03-25

**Authors:** Johan Coenen, Bart H. H. Golsteyn, Tom Stolp, Dirk Tempelaar

**Affiliations:** School of Business and Economics, Maastricht University, Maastricht, The Netherlands; IZA - Institute of Labor Economic, GERMANY

## Abstract

In this study, we investigate whether Conscientiousness, Emotional Stability and Risk Preference relate to student performance in higher education. We employ anchoring vignettes to correct for heterogeneous scale use in these non-cognitive skills. Our data are gathered among first-year students at a Dutch university. The results show that Conscientiousness is positively related to student performance, but the estimates are strongly biased upward if we use the uncorrected variables. We do not find significant relationships for Emotional Stability but find that the point estimates are larger when using the uncorrected variables. Measured Risk Preference is negatively related to student performance, yet this is fully explained by heterogeneous scale use. These results indicate the importance of using more objective measurements of personality traits.

## 1. Introduction

Personality is an important predictor of life outcomes (see, e.g., [1, 2]). Personality is typically measured by asking individuals to evaluate self-reflective statements on subjective scales. One important issue with this approach is that people may systematically differ in scale use. If differences in scale use are correlated to outcomes, the relationship between measures of personality traits and such outcomes can be biased.

In this paper, we study whether people differ systematically in the values they attach to personality item scales and whether these systematic differences bias the relationship between measured personality traits and academic performance. We study biases with respect to Emotional Stability, Conscientiousness, and Risk Preference. The first two are personality traits from the Big Five Inventory. Risk Preference is an economic preference parameter and not a personality trait. However, for the sake of brevity, we refer in this paper to Conscientiousness, Emotional Stability, and Risk Preference as “personality.”

Scale use biases the relationship between self-reported personality and academic performance if it is related both to self-reported personality and to academic performance. The relationship between scale use and self-reported personality is obvious. The relationship between scale use and academic performance can occur, for instance, if students who score higher on achievement tests have different comparison groups in mind when completing the survey on personality traits than students who score lower on achievement tests.

To separate true differences in personality from differences in scale use, we employ anchoring vignettes (see [3, 4]). We first ask questions to measure Conscientiousness, Emotional Stability, and Risk Preference. Then, we ask the same questions for a fictive hairdresser, surgeon, firefighter, and bus driver. We use the answers on the latter questions to identify heterogeneity in scale use. In a final step, we analyze whether correlations with academic performance differ for the uncorrected and corrected answers on personality traits.

The main result of our analysis is that the relationship between uncorrected personality and academic outcomes is overestimated if differences in scale use are not taken into account. This conclusion holds for all traits we investigate.

There is a vast literature on the relationship between personality traits and academic performance. For an overview, see, e.g., [1, 5–7]. Fig 3 in [1] (p. 1007) summarizes some of the main conclusions from this literature; of the Big Five traits, Conscientiousness is most strongly related to college grades. There is no strong correlation between Emotional Stability and grades. There is no consensus in the literature concerning the relationship between Risk Preference and academic performance (see the overviews in [1, 8]).

Our paper contributes to the literature which studies whether answers on subjective scales differ from objectively measured indicators. For instance [9], investigate why workers in different Western countries report very different rates of work disability. They show that Dutch respondents have a lower threshold in reporting whether they have a work disability than American respondents [10] investigate the difference between stated physical activity and physical activity measured by accelerometers across different countries. The self-reported data show minor differences across countries while the accelerometer data show that the Dutch and English appeared to be much more physically active than Americans. Regarding life satisfaction [11], show that Americans are more likely to use the extremes of the scale than the Dutch, who are more inclined to stay in the middle of the scale.

As proposed in [12], an important challenge in personality psychology is finding objective measures or vignettes for personality traits to correct for bias due to scale use. Some previous research has applied anchoring vignettes to measure personality traits more accurately. [13] show that self-reports of non-cognitive skills are sensitive to survey administration conditions. Providing information about the importance of non-cognitive skills to students, for instance, directly affects their responses. [14] use anchoring vignettes to compare measures of Conscientiousness across 21 countries and show that country rankings of self-reported Conscientiousness to some degree result from differences in response styles. [15] show that the reliability of scales assessing Conscientiousness and Openness to Experience increases when using anchoring vignettes in a study of 12th-grade students in Brazil. [16] employ anchoring vignettes for the Big Five Inventory in Rwanda and the Philippines. They show that adjusted scores have better measurement properties relative to scores based on the original Likert scale. In their study, correlations of the Big Five personality factors with life satisfaction were essentially unchanged after the vignette-adjustment while correlations with counterproductive behavior were noticeably lower. We contribute to this literature by focusing on the relationship between personality traits and academic performance.

Our findings have important policy implications. The predictive power of personality and preferences for academic achievement found in earlier research may serve as a tool for policy makers. Personality and preferences can serve as signals for future performance and, therefore, policy makers can help children with adverse personality traits or preferences, e.g. by providing more support to such children. Virtually all papers studying the relationship between personality and achievement have used subjective measures of personality. Our paper indicates that the predictive power of subjective measures of personality may be overestimated. The correlations between personality and academic performance will be more useful for policy makers if more objective measures of personality traits are employed.

The set-up of this paper is as follows. Section 2 discusses the data. Section 3 shows the empirical strategy. Section 4 reports the results. Section 5 concludes.

## 2. Data

### 2.1. Ethics statement

Ethics approval was obtained by the Ethical Review Committee Inner City faculties of Maastricht University (ERCIC_044_14_07). Participants of the research all provided written consent.

### 2.2. Sample

We collected data among students at a Dutch Economics and Business school in the first course of the first year. In the Netherlands, curricula of Economics and Business programs typically share several introductory courses, giving rise to classes counting 1000 students or more. The data collection consisted of two parts. The first was held as a mandatory assignment in an introductory course in quantitative methods. This implied that all active students participated in the survey. In this first part, questions about Risk Preference were included. As a result, we have information on Risk Preference of 1056 students. We also have information on the grade obtained in this course of all these students. Risk Preference was measured twice: in week 1 and week 6 of the seven-weeks course. Because we measure Risk Preference vignettes in week 6, we also use data on Risk Preference from week 6. Using the data on Risk Preference from week 1, however, yields very similar results. This implies that Risk Preference remains relatively stable during this education period (the correlation between the measures is 0.555, p<0.0000) and therefore, that reverse causality does not appear to play a role. In the analyses on Risk Preference, we furthermore restrict the sample to those who also answered the questions on Conscientiousness and Emotional Stability in order to show estimations on the same sample for all traits. If we do not restrict the sample, we again find very similar results.

A second survey was held one week after the course ended. Participation in this survey was voluntary. We gave every fifth participant 10 euros and held a lottery among all participants with a 1000 euros prize. In total, 625 students participated. We have information about these students on their Conscientiousness and Emotional Stability.

Table A in S1 File, presents summary statistics on the differences between respondents who participated in the first and second survey and respondents who participated in the first but not the second survey. For the observables we investigate, the groups appear to be comparable although grades are significantly higher in the in-sample and there are slightly more women in the in-sample than in the out of sample group. Note that we cannot compare the level of Conscientiousness or Neuroticism between these two groups since this information was only collected in the second survey.

### 2.3. Measuring personality

We follow the non-parametric approach developed by [3] and in more detail described by [4]. In their non-parametric approach, they use anchoring vignettes to adjust for respondents’ scale use. In this way, it is possible to recode variables such that respondents’ answers are on the same scale.

We first measure personality of a respondent. For Conscientiousness, we pose the statement: “I am always prepared.” For Emotional Stability, we pose the statement “I get stressed out easily.” For Risk Preference, we ask: “Are you generally willing to take risks, or do you try to avoid risks?” Response categories on all statements and questions range from 1 “I fully disagree” to 7 “I fully agree.” The question we use to measure Risk Preference is taken from the German Socio-Economic Panel and validated by [17]. They show that responses to the survey question predict behavior in incentivized choices under risk. The items we use to measure Conscientiousness and Emotional Stability are taken from standard questionnaires to measure Big Five constructs [18]. In personality psychology, it is common to use more than one question to measure a trait. However, it is important to assess the differences in scale use for personality items separately. Different items may induce different response mechanisms. The wording of items can evoke different interpretations between groups, for example. Grouping items together to reduce measurement error presupposes a common error structure (e.g., all measurement error is independent) of the items. In this paper, we argue the opposite and focus on the item-level to analyze scale use that is specific to the item. As such, we need a vignette for each question we pose. Due to space limitations in the survey, we can therefore only include one item to measure the trait.

Secondly, we ask the same questions, but for fictive persons. For instance, for Conscientiousness, we state the following: “Imagine a surgeon. To what extent does the following statement apply: A surgeon is always prepared.” We have two vignettes for this item of Conscientiousness: “A hairdresser/surgeon is always prepared.” For Emotional Stability, we have two vignettes on one item of the trait: “A hairdresser/surgeon gets stressed out easily.” For Risk Preference, we have three vignettes: “Is a bus driver/firefighter/hairdresser generally willing to take risks, or does (s)he try to avoid risks?”

We then use the answers for the fictive persons as anchoring vignettes for the answers on the subjective question about oneself. The idea is that the way in which subjects report about their own latent trait coincides with the way they report about the latent trait of a “generic” other (e.g. hairdresser). For example, individuals may differ in their interpretation of the trait or answer categories as described in the self-assessment. As the vignette contains the same description of the trait, the vignette presumably captures the same scale use as the self-assessment. In addition, the tendency to agree with statements independent of their content–i.e. acquiescence bias–is captured by both types of assessments. Moreover, the use of vignettes allows us to correct for reference bias. In particular, an individual may evaluate his or her personality in comparison to a reference group (e.g., those who are close like friends, family and colleagues) such that the response portrays an individual’s stance within the reference group. If personality traits in the group are correlated, then someone might report a low score even though they have a high value at the population-level. By introducing a vignette, such errors are corrected.

To do so, we assume a logical order of the vignettes. In the case of Conscientiousness, we assume that surgeons are more conscientious than hairdressers. For the respondents who rated the vignettes in this logical order, we can correct the personality trait by recoding the variable in the following way: C = 1 if y < v1; C = 2 if y = v1; C = 3 if v1 < y < v2; C = 4 if v1 < y = v2; C = 5 if y > v2, where y is the respondents’ self-assessment on the personality trait, v_1_ is the response for the same respondent for a fictive hairdresser and v_2_ is the response for the fictive surgeon. For Emotional Stability, we assume that a surgeon is emotionally more stable than a hairdresser. For Risk Preference, we assume that a firefighter is more willing to take risks than a bus driver.

In principle, it is an interesting question whether surgeons are observed to be on average more conscientious/emotionally stable. However, for our paper, this is not very important. Crucial for our analyses is that students perceive surgeons to be more conscientious/emotionally stable than hairdressers, or at least they perceive themselves to be on a different level of Conscientiousness/Emotional Stability than those occupations, because the vignettes are mainly about putting everyone’s answers on the same scale. The following percentage of the students indeed do perceive the surgeons to be more conscientious/emotionally stable than the hairdressers: 79.6% of the sample (Conscientiousness) and 56.2% (Emotional Stability). When we allow for a different ordering, these percentages increase to: 94.8% of the sample (Conscientiousness) and 86.0% (Emotional Stability).

Perceptions of the vignettes may differ depending on the occupation of the respondent. E.g., a hairdresser might perceive a hairdresser’s Conscientiousness differently than a surgeon. These influences may be somewhat weaker in the sample, as the respondents are economics students. This firstly means that they are very much alike such that they would all suffer the same “misperception.” Differences between students can then still be interpreted as differences in scale use. Secondly, because we focus on first-year students of Economics and Business, these respondents are not in an occupation yet, besides some small jobs on the side, which are very unlikely to include hairdressing, let alone work as a surgeon. Therefore, we expect that the vast majority of the students will indeed consider service occupations and medical occupations in general.

Respondents do not always follow the intended rank of the vignettes: i.e., either they tie vignettes (e.g., hairdressers are equally conscientious as surgeons) or they misplace them (e.g., surgeons are less conscientious as hairdressers). This leads to the 13 options in Table 1. For Risk Preference, we show two sets of two vignettes in this table: (A) bus driver and firefighter; and (B) hairdresser and firefighter. Alternatively, we can use all three vignettes, but we do not show this in Table 1 since using three vignettes yields 75 ordering options.

**Table 1:** The distribution of all options of self-assessment and vignette ratings.

			Conscientiousness	Emotional Stability	Risk Preference	
	Ordering	New rating	Always prepared	Get stressed easily (Reversed)	A	B
1	y < v1 < v2	1	23.0	22.99	4.79	7.62
2	y = v1 < v2	2	18.7	8.37	6.61	10.10
3	v1 < y < v2	3	27.8	12.97	65.95	59.77
4	v1 < y = v2	4	8.4	9.36	12.56	12.75
5	v1 < v2 < y	5	2.8	2.96	4.46	3.48
6	y < v1 = v2	1	13.0	15.76	0.33	0.33
7	y = v1 = v2	{2,3,4}	1.6	3.45	1.32	1.16
8	v1 = v2 < y	5	0.8	2.3	1.49	0.83
9	y < v2 < v1	1	1.3	10.67	0.17	0.00
10	y = v2 < v1	{1,2,3,4}	0.8	4.76	0.17	0.17
11	v2 < y < v1	{1,2,3,4,5}	0.5	2.96	0.33	0.83
12	v2 < y = v1	{2,3,4,5}	0.8	1.81	0.50	0.83
13	v2 < v1 < y	5	0.5	1.64	1.32	2.15
% completely correct ordering			80.62	56.65	94.38	93.71
% Without intervals			96.22	87.03	97.69	97.02
N			609	609	605	604

Note: Risk Preference A uses bus driver as vignette 1. Risk Preference B uses hairdresser as vignette 1. In both cases, vignette 2 is firefighter. y = own value on personality trait; v1 = value on vignette 1 (hairdresser for Conscientiousness, Emotional Stability and Risk Preference B, bus driver for Risk Preference A); v2 = value on vignette 2 (surgeon for Conscientiousness and Emotional Stability, firefighter for Risk Preference A and B). Percentage “completely correct ordering” is the share of the sum of rows 1–5 relative to the total number of respondents. Percentage “without intervals” is the share of the sum of rows 1–5, 6, 8, 9, and 13. Row 7 shows the case when respondents rate themselves the same as both vignettes. The only thing one can conclude from this is that the new rating of the respondent will definitely not be lower (1) or higher (5) than both vignettes. It is, however, impossible to distinguish between the values 2, 3 and 4, because we cannot put the own value lower or higher than any of the vignettes. In the case of row 10, we only know that the respondents have rated themselves lower than vignette 1, and the same as vignette 2. This only excludes the value 5. Row 12 is the mirrored image of this. Row 11 is the option which gives no information at all because there are both no ‘ties’ and the ordering of the vignettes is wrong.

The bottom rows of the table show how many respondents order vignettes in a logical way. Using the strictest definition of logical order, 80.6% of the respondents ordered the vignettes for the Conscientiousness item “Always prepared” in a logical way. For Risk Preference, both sets of vignettes are ordered logically by almost all respondents (94.4% for set A and 93.4% for set B). For Emotional Stability, we find that fewer respondents logically order their answers. A reason may be that some respondents do not think about a person when answering the questions but about a profession. So, they think for instance, about surgeons working in a stressful environment instead of that a surgeon may be more stress-resistant.

This rescaling technique uses the ordering of the answer relative to the vignettes, which is more objective than the answers on the subjective question. The anchoring corrected personality trait has 5 values: the value 1 if row 1 applies, 2 if row 2 applies, etc.

Besides the most logical ordering of the first five rows, rows 6, 8, 9, and 13 may also be plausible answers since the own value and the vignettes are also separable in these rows. In rows 6 and 8, respondents value the vignettes similarly, but because the own value is above (below) both vignettes, one can still conclude that the own value is lower (higher) than both vignettes. Therefore, for these rows, we can scale the anchoring corrected variable to 1 (5). In rows 9 and 13, the vignettes have been reversed by the respondent, but because the own value is lower (higher) than both vignettes, one can conclude that this is lower (higher) than both vignettes, and therefore we can rescale the answer to 1 (5).

Using this additional insight, we define two samples. The baseline sample contains only respondents with answers 1–5, and the extended sample additionally includes rows 6, 8, 9, and 13. All other answers are less plausible and will be excluded from the analyses.

Tables B-D in S1 File, give summary statistics of the samples for respectively Conscientiousness, Emotional Stability, and Risk Preference.

## 3. Empirical strategy

### 3.1. Subjective and anchoring vignette corrected measures of personality

We first estimate the following type of functions:

$$P_{i}=X_{i}\delta^{\prime }+\varepsilon_{i},$$

in which *P_i_* is the personality trait: Conscientiousness, Emotional Stability, or Risk Preference of individual *i*. *X_i_* indicates a set of explanatory variables: math major, math entry test score, female, age, parental education, nationality (Dutch (reference group), Belgian, German, other nationality), and study field (Economics and Business (reference group), Fiscal Economics, International Business). The relationship of these variables with personality traits is captured by the vector *δ*′. The error term is denoted as *ε_i_*.

We run this type of regressions separately for subjective personality traits, and for anchoring vignette corrected personality measures. We do this both for the baseline and the extended sample. We make use of ordered probit models due to the ordinal nature of the dependent variable. The results indicate if relationships between subjective personality and explanatory variables are robust when using the anchoring vignette corrected personality measures.

### 3.2. Relation between grade and personality

Our main analyses are given by the function:

$$G_{i}=P_{i}\beta^{\prime }+X_{i}\alpha^{\prime }+\mu_{i},$$

in which *G_i_* is the grade obtained in the quantitative methods course. *β*′ measures the effect of personality traits on this grade. The relationship of background variables with personality traits is captured by the vector *α*′. The error term is denoted as *μ_i_*. Similar to the first equation, we estimate this function separately for the three personality traits, which are in turn measured subjectively and anchoring vignette corrected respectively, and we do this both for the baseline and extended sample. We use OLS regressions with fixed effects for the student’s study program. The distribution of the dependent variable Grade is between 1–10 with steps of 0.5-grade point. With 19 different options, we see OLS as best suited in this case. The results indicate if relationships between grades and subjective personality measures are robust when using the anchoring vignette corrected personality measures.

## 4. Results

The first part of the empirical results shows whether the student characteristics are related differently to corrected and uncorrected personality measures. Tables 2–4 give the results of two regressions: One between the uncorrected personality measures and characteristics X′, and another between the corrected personality measures and X′. By doing so, we follow [3] and [9] who show that background characteristics explain both the latent trait and scale use. It follows that scale use can differ structurally between individuals.

**Table 2:** Conscientiousness and background characteristics.

	Ordered probit	Baseline sample	Vignette baseline	Extended sample	Vignette extended
Math Major	0.173	0.157	0.146	0.165	0.229*
	(0.093)	(0.105)	(0.108)	(0.095)	(0.099)
Math entry test	0.003	-0.002	0.003	-0.000	-0.003
	(0.014)	(0.017)	(0.017)	(0.015)	(0.016)
Female	0.443***	0.480***	0.418***	0.488***	0.293**
	(0.088)	(0.100)	(0.102)	(0.091)	(0.094)
Age	-0.016	-0.052	0.022	-0.030	-0.025
	(0.034)	(0.041)	(0.042)	(0.035)	(0.037)
Parental education: university	-0.084	-0.144	-0.021	-0.072	-0.044
(ref.: lower than university)	(0.104)	(0.120)	(0.124)	(0.108)	(0.113)
Belgian	0.013	0.067	0.093	-0.011	-0.020
	(0.144)	(0.163)	(0.168)	(0.148)	(0.156)
German	0.313*	0.303*	0.095	0.243	0.252
	(0.126)	(0.143)	(0.148)	(0.132)	(0.138)
Other nationality	-0.083	-0.025	-0.150	-0.181	-0.218
(ref.: Dutch)	(0.135)	(0.156)	(0.162)	(0.140)	(0.149)
Study: Fiscal Economics	0.441	0.554*	0.684*	0.397	0.522*
(Ref.: Economics and Business)	(0.254)	(0.277)	(0.281)	(0.256)	(0.262)
International Business	-0.088	-0.062	-0.056	-0.147	-0.138
	(0.092)	(0.104)	(0.108)	(0.095)	(0.100)
Observations	594	473	473	563	563
Pseudo R-squared	0.023	0.025	0.021	0.026	0.021

Note: Each column reports a regression with Conscientiousness as the dependent variable (standardized to a mean of zero and standard deviation 1) and the variables in the rows as the independent variables. The baseline sample contains only respondents with answers 1–5 of Table 1, and the extended sample additionally includes rows 6, 8, 9, and 13. Standard errors are reported in parentheses

*** p<0.001

** p<0.01

* p<0.05.

**Table 3:** Emotional Stability and background characteristics.

	Ordered probit	Baseline sample	Vignette baseline	Extended sample	Vignette extended
Math Major	0.177	0.197	0.252	0.172	0.323**
	(0.092)	(0.123)	(0.130)	(0.100)	(0.112)
Math entry test	0.012	0.010	0.012	0.013	0.001
	(0.014)	(0.020)	(0.021)	(0.015)	(0.018)
Female	-0.639***	-0.580***	-0.583***	-0.661***	-0.624***
	(0.088)	(0.122)	(0.132)	(0.096)	(0.110)
Age	0.049	0.082	0.070	0.049	0.043
	(0.034)	(0.044)	(0.047)	(0.035)	(0.039)
Parental education: university	0.083	-0.029	0.040	0.132	0.071
(ref.: lower than university)	(0.103)	(0.138)	(0.147)	(0.113)	(0.127)
Belgian	-0.606***	-0.524**	-0.392	-0.653***	-0.427*
	(0.145)	(0.195)	(0.207)	(0.157)	(0.177)
German	-0.124	-0.057	-0.169	-0.144	-0.171
	(0.125)	(0.156)	(0.164)	(0.134)	(0.146)
Other nationality	-0.396**	-0.085	-0.160	-0.389**	-0.454**
(ref.: Dutch)	(0.135)	(0.177)	(0.187)	(0.146)	(0.164)
Study: Fiscal Economics	-0.232	-0.659*	-0.785*	-0.361	-0.184
(Ref.: Economics and Business)	(0.251)	(0.327)	(0.368)	(0.264)	(0.296)
International Business	0.056	-0.023	0.081	0.047	0.081
	(0.092)	(0.124)	(0.132)	(0.099)	(0.112)
Observations	594	334	334	511	511
Pseudo R-squared	0.037	0.032	0.036	0.039	0.041

Note: Each column reports a regression with Emotional Stability as the dependent variable (standardized to a mean of zero and standard deviation 1) and the variables in the rows as the independent variables. The baseline sample contains only respondents with answers 1–5 of Table 1, and the extended sample additionally includes rows 6, 8, 9, and 13. Standard errors are reported in parentheses

*** p<0.001

** p<0.01

* p<0.05.

**Table 4:** Risk Preference and background characteristics.

	Ordered probit	Baseline sample	Vignette baseline	Extended sample	Vignette extended
Math Major	-0.102	-0.134	0.044	-0.103	0.032
	(0.095)	(0.099)	(0.108)	(0.096)	(0.104)
Math entry test	0.010	0.018	0.022	0.012	0.010
	(0.015)	(0.016)	(0.017)	(0.015)	(0.016)
Female	-0.472***	-0.430***	-0.370***	-0.468***	-0.373***
	(0.090)	(0.093)	(0.102)	(0.091)	(0.098)
Age	0.025	0.037	0.038	0.021	0.048
	(0.035)	(0.036)	(0.039)	(0.035)	(0.038)
Parental education: university	-0.033	-0.051	0.010	-0.055	-0.024
(ref.: lower than university)	(0.106)	(0.110)	(0.121)	(0.108)	(0.117)
Belgian	0.163	0.205	-0.183	0.183	-0.030
	(0.148)	(0.153)	(0.168)	(0.150)	(0.162)
German	0.034	0.058	-0.183	0.081	-0.092
	(0.128)	(0.133)	(0.146)	(0.131)	(0.143)
Other nationality	0.477***	0.495***	0.064	0.503***	0.150
(ref.: Dutch)	(0.139)	(0.143)	(0.156)	(0.142)	(0.153)
Study: Fiscal Economics	-0.290	-0.280	-0.319	-0.265	-0.279
(Ref.: Economics and Business)	(0.267)	(0.269)	(0.304)	(0.268)	(0.299)
International Business	0.065	0.057	0.060	0.073	0.097
	(0.094)	(0.097)	(0.106)	(0.095)	(0.103)
Observations	583	548	548	568	568
Pseudo R-squared	0.026	0.026	0.021	0.026	0.018

Note: Each column reports a regression with grades as the dependent variable (standardized to a mean of zero and standard deviation 1) and the variables in the rows as the independent variables. The baseline sample contains only respondents with answers 1–5 of Table 1, and the extended sample additionally includes rows 6, 8, 9, and 13. Standard errors are reported in parentheses

*** p<0.001

** p<0.01

* p<0.05.

In the tables, column 1 shows the results for the full sample. We focus on comparing results of corrected and uncorrected measures of the personality traits in the baseline (columns 2 and 3) and the extended sample (columns 4 and 5). Regarding the results for Emotional Stability (Table 3), for instance, Column 2 shows that Belgian students report lower scores of Emotional Stability than Dutch students in the baseline sample (given other characteristics). However, column 3 reveals that this relationship is not robust when using the corrected measure for Emotional Stability. In the extended sample, we also find that this relationship is less strong when using the corrected measure than when we use the uncorrected measure.

These results are important as they reveal that people differ in their scale use systematically. For example, nationality may affect how you interpret the scale or the text that describes traits and behaviors. If such systematic differences exist, it becomes questionable whether the relationship between uncorrected personality measures and outcomes such as academic performance represents the true underlying relationship. Academic performance in itself may alter one’s scale use, for example, such that the linear relation is under- or overestimated. Moreover, any other covariates used to predict academic performance may yield biased estimates as they covary both with the latent trait and scale use. In sum, showing that people differ in scale use depending on their background characteristics provides evidence that using uncorrected measures as predictors may be unwarranted.

Tables 5–7 report the relationships between grades and personality traits. For instance, Table 7, column 2, shows that willingness to take risks appears to be strongly related to grades. One standard deviation higher willingness to take risks is related to a grade around 0.26 points lower on a scale of 1–10. However, column 3 reveals that the relationship is no longer significant when using the corrected measure of Risk Preference. Therefore, there is a large overestimation (i.e., in absolute sense) of the relationship of Risk Preference and grades if the vignette is not used. These results also hold if we use the extended sample.

**Table 5:** Relationship between Conscientiousness and grades.

	Original	Baseline sample	Vignette baseline	Extended sample	Vignette extended
I am always prepared	0.303***	0.347***	0.165	0.337***	0.202*
	(0.080)	(0.089)	(0.089)	(0.083)	(0.082)
Math Major in high school	1.251***	1.230***	1.257***	1.252***	1.261***
	(0.167)	(0.186)	(0.189)	(0.171)	(0.173)
Math entry test	0.146***	0.131***	0.131***	0.145***	0.146***
	(0.026)	(0.030)	(0.030)	(0.027)	(0.027)
Female	-0.108	-0.098	-0.013	-0.117	-0.025
	(0.160)	(0.179)	(0.180)	(0.165)	(0.164)
Age	-0.049	0.007	-0.014	-0.034	-0.039
	(0.062)	(0.073)	(0.074)	(0.063)	(0.063)
Parental education: university	0.084	0.046	0.001	0.106	0.089
	(0.187)	(0.214)	(0.216)	(0.194)	(0.196)
Belgian	-0.867***	-0.799**	-0.793**	-0.877**	-0.875**
	(0.261)	(0.290)	(0.294)	(0.267)	(0.270)
German	-0.232	-0.238	-0.155	-0.212	-0.183
	(0.229)	(0.255)	(0.257)	(0.238)	(0.240)
Other nationality	-1.193***	-1.032***	-1.016***	-1.080***	-1.097***
	(0.245)	(0.279)	(0.282)	(0.253)	(0.255)
Constant	5.967***	5.045***	5.396***	5.632***	5.680***
	(1.237)	(1.460)	(1.478)	(1.256)	(1.268)
Observations	594	473	473	563	563
Adjusted R-squared	0.211	0.196	0.176	0.212	0.197
% of sample because of vignette ordering			79.63		94.78

Note: Each column reports a regression with grades as the dependent variable and the variables in the rows as the independent variables. Conscientiousness (“I am always prepared”) is standardized to a mean of zero and standard deviation 1. The baseline sample contains only respondents with answers 1–5 of Table 1, and the extended sample additionally includes rows 6, 8, 9, and 13. Standard errors are reported in parentheses

*** p<0.001

** p<0.01

* p<0.05.

**Table 6:** Relationship between Emotional Stability and grades.

	Original	Baseline sample	Vignette baseline	Extended sample	Vignette extended
I get stressed out easily (reversed)	0.168*	0.174	0.065	0.157	0.044
	(0.083)	(0.106)	(0.105)	(0.088)	(0.087)
Math Major in high school	1.269***	1.306***	1.325***	1.281***	1.296***
	(0.169)	(0.215)	(0.216)	(0.180)	(0.181)
Math entry test	0.146***	0.166***	0.167***	0.157***	0.159***
	(0.026)	(0.035)	(0.035)	(0.028)	(0.028)
Female	0.110	0.073	0.016	0.233	0.161
	(0.165)	(0.218)	(0.218)	(0.178)	(0.176)
Age	-0.061	0.021	0.029	-0.057	-0.053
	(0.062)	(0.078)	(0.078)	(0.064)	(0.064)
Parental education: university	0.048	-0.104	-0.110	-0.127	-0.112
	(0.189)	(0.242)	(0.242)	(0.203)	(0.203)
Belgian	-0.772**	-0.422	-0.483	-0.730*	-0.804**
	(0.267)	(0.341)	(0.341)	(0.284)	(0.282)
German	-0.125	0.023	0.025	-0.016	-0.028
	(0.230)	(0.273)	(0.275)	(0.242)	(0.243)
Other nationality	-1.153***	-0.868**	-0.872**	-1.143***	-1.182***
	(0.249)	(0.311)	(0.312)	(0.265)	(0.266)
Constant	6.045***	4.349**	4.262**	5.945***	5.895***
	(1.248)	(1.560)	(1.567)	(1.288)	(1.291)
Observations	594	334	334	511	511
Adjusted R-squared	0.197	0.212	0.206	0.213	0.209
% of sample because of vignette ordering			56.23		86.03

Note: Each column reports a regression with grades as the dependent variable and the variables in the rows as the independent variables. Emotional Stability (the reverse of “I get stressed out easily”) is standardized to a mean of zero and standard deviation 1. The baseline sample contains only respondents with answers 1–5 of Table 1, and the extended sample additionally includes rows 6, 8, 9, and 13. Standard errors are reported in parentheses

*** p<0.001

** p<0.01

* p<0.05.

**Table 7:** Relationship between Risk Preference and grades.

	Original	Baseline sample	Vignette baseline	Extended sample	Vignette extended
Are you generally willing to take risks?	-0.197*	-0.257**	-0.034	-0.249**	-0.065
	(0.077)	(0.079)	(0.079)	(0.078)	(0.078)
Math Major in high school	1.207***	1.188***	1.224***	1.198***	1.227***
	(0.167)	(0.171)	(0.173)	(0.168)	(0.169)
Math entry test	0.163***	0.175***	0.171***	0.168***	0.165***
	(0.026)	(0.027)	(0.027)	(0.026)	(0.026)
Female	-0.105	-0.063	0.017	-0.111	-0.035
	(0.159)	(0.161)	(0.162)	(0.160)	(0.160)
Age	-0.045	-0.027	-0.029	-0.045	-0.042
	(0.061)	(0.063)	(0.063)	(0.062)	(0.062)
Parental education: university	0.070	0.082	0.092	0.097	0.106
	(0.186)	(0.191)	(0.193)	(0.188)	(0.190)
Belgian	-0.846**	-0.859**	-0.922***	-0.872***	-0.923***
	(0.260)	(0.266)	(0.268)	(0.262)	(0.263)
German	-0.096	-0.050	-0.064	-0.100	-0.118
	(0.226)	(0.230)	(0.233)	(0.230)	(0.232)
Other nationality	-1.109***	-1.015***	-1.137***	-1.067***	-1.182***
	(0.246)	(0.249)	(0.248)	(0.249)	(0.248)
Constant	5.733***	5.270***	5.339***	5.680***	5.643***
	(1.226)	(1.258)	(1.271)	(1.233)	(1.245)
Observations	583	548	548	568	568
Adjusted R-squared	0.208	0.224	0.209	0.219	0.206
% of sample because of vignette ordering			94.00		97.43

Note: Each column reports a regression with grades as the dependent variable and the variables in the rows as the independent variables. Risk Preference (“Are you generally willing to take risks, or do you try to avoid risks?”) is standardized to a mean of zero and standard deviation 1. The baseline sample contains only respondents with answers 1–5 of Table 1, and the extended sample additionally includes rows 6, 8, 9, and 13. Standard errors are reported in parentheses

*** p<0.001

** p<0.01

* p<0.05.

Tables 5 and 6 show the results for Conscientiousness and Emotional Stability. Comparing columns 2 and 3 in these tables reveals a similar pattern as we find for Risk Preference; the point estimates of the relationships between these personality traits and grades are much larger if the vignette is not used than if it is used.

In this analysis, we allow “incorrect” orderings by students to be included. In Tables 5–7 we compare (a) the original Conscientiousness/Emotional Stability/Risk Preference measure for all respondents, (b) the original Conscientiousness/Emotional Stability/Risk Preference measure for those respondents with the correct ordering on the vignette (Conscientiousness: 79.6% of the sample; Emotional Stability: 56.2%; Risk Preference: 94.0%), (c) the new vignette-adjusted measure of Conscientiousness/Emotional Stability/Risk Preference for those respondents with the correct ordering on the vignette (Same proportion as (b)), (d) the original Conscientiousness/Emotional Stability/Risk Preference measure for the respondents from (b) plus those respondents with a more lenient ordering on the vignette (‘mistakes’ are allowed as long as a clear separation between own answer and the vignettes can still be made; Conscientiousness: 94.8% of the sample; Emotional Stability: 86.0%; Risk Preference: 97.4%), and (e) the new vignette-adjusted measure of Conscientiousness/Emotional Stability/Risk Preference for those respondents with the correct ordering on the vignette plus those respondents with a more lenient ordering on the vignette (Same proportion as (d)).

The results remain robust independent of the usage of the baseline sample or the extended sample, with the exception of Conscientiousness, for which the coefficient becomes somewhat larger and the p-value turns below 0.05 (reassuringly, the two coefficients are not statistically different from each other).

## 5. Conclusions

This paper investigates the relationship between student performance in higher education and Conscientiousness, Emotional Stability and Risk Preference. We employ anchoring vignettes to correct for heterogeneous scale use in these non-cognitive skills. Our main result is that if scale use is not taken into account, the relationship between academic achievement and personality traits is overestimated. This holds for all traits we investigated.

Our results have important implications for the literature studying the relationship between personality and outcomes such as school achievement. Previous research has shown strong correlations between personality and such outcomes, but our paper suggests that these correlations may be overestimated. Using vignettes or more objective personality measures is important to get unbiased estimates of the predictive power of personality for outcomes in life.

One of our research limitations is that our sample of college students is not representative of the full Dutch population. Future research is needed to see if the results are robust in representative samples. A second limitation is that the anchors we use may in themselves also be subject to bias. For instance, students who have hairdressers in their direct environment may judge hairdressers’ personality traits to be different from students who do not have hairdressers in their direct environment. Moreover, having a hairdresser in the direct environment may be related to their academic outcomes. In this case, the relationship between corrected traits and academic outcomes may be biased. We control for parental education levels so this issue may not lead to much bias in our estimations, but the example does point out that future work needs to focus on finding anchors which are more objective than ours and are not susceptible to structural differences in vignette perception. Finding the perfect anchors has not been our focus. Our work, instead, serves as a first step in investigating the extent to which using more objective measures of personality traits influences the estimated relationships between traits and outcomes.

## Supporting information

S1 File Appendix. (DOCX)
