# Supplementary material for: Personality traits and academic performance: Correcting self-assessed traits with vignettes
Source: PLoS One. 2021 Mar 25;16(3):e0248629. doi: 10.1371/journal.pone.0248629 (PMC7993818; doi:10.1371/journal.pone.0248629)
Supplement: S1 File — (DOCX) [file pone.0248629.s001.docx]

**S1 file. Appendix**

**Table A. Differences in means between the estimation sample and the full sample**

|  | In sample | Out of sample | Difference |
| --- | --- | --- | --- |
| Final grade QM1 | 6.16 | 5.63 | -0.53*** |
| Math Major in high school | 0.40 | 0.35 | 0.06 |
| Math entry test | 0.43 | 0.41 | 0.02 |
| Gender: female | 0.45 | 0.37 | 0.08** |
| Age: 19 or older | 0.50 | 0.54 | -0.04 |
| Dutch | 0.22 | 0.26 | -0.03 |
| Belgian | 0.17 | 0.17 | -0.00 |
| German | 0.35 | 0.37 | -0.01 |
| Other nationality | 0.25 | 0.20 | 0.05 |

Note: Column 1 shows the means of the variables in the estimation sample. Column 2 shows the means of the respondents of the first survey who did not respond to the second survey. Parental education was measured in the second survey, so we cannot do a comparison for this variable. *** p<0.001, ** p<0.01, * p<0.05.

**Table B. Summary statistics Conscientiousness**

|  | No | | | Yes | | | Difference | | |
| --- | --- | --- | --- | --- | --- | --- | --- | --- | --- |
|  | Original | Vignette baseline | Vignette extended | Original | Vignette baseline | Vignette extended | Original | Vignette baseline | Vignette extended |
|  |  |  |  |  |  |  |  |  |  |
| Female | 4.42 | 2.22 | 2.09 | 4.92 | 2.59 | 2.36 | -0.50*** | -0.37*** | -0.27** |
|  |  |  |  |  |  |  |  |  |  |
| Age: 19 and older | 4.61 | 2.34 | 2.18 | 4.68 | 2.40 | 2.23 | -0.07 | -0.05 | -0.05 |
|  |  |  |  |  |  |  |  |  |  |
| Parents higher education | 4.73 | 2.45 | 2.28 | 4.61 | 2.34 | 2.19 | 0.12 | 0.11 | 0.09 |
|  |  |  |  |  |  |  |  |  |  |
| Nationality: |  |  |  |  |  |  |  |  |  |
| Dutch | 4.66 | 2.36 | 2.20 | 4.57 | 2.39 | 2.22 | 0.09 | -0.03 | -0.02 |
| Belgian | 4.66 | 2.36 | 2.22 | 4.54 | 2.44 | 2.15 | 0.12 | -0.08 | 0.07 |
| German | 4.52 | 2.34 | 2.11 | 4.87 | 2.42 | 2.39 | -0.34** | -0.08 | -0.28** |
| Other | 4.71 | 2.42 | 2.28 | 4.46 | 2.22 | 1.99 | 0.24* | 0.20 | 0.29** |
|  |  |  |  |  |  |  |  |  |  |
| Math Major in SE | 4.59 | 2.32 | 2.12 | 4.72 | 2.42 | 2.30 | -0.14 | -0.10 | -0.18 |
|  |  |  |  |  |  |  |  |  |  |
| Math entry test: 8-14 | 4.64 | 2.34 | 2.20 | 4.63 | 2.36 | 2.14 | 0.02 | -0.02 | 0.06 |
|  |  |  |  |  |  |  |  |  |  |

Note: “No” indicates the value 0 and “Yes” implies the value 1 for the variables displayed in the rows. The baseline sample contains only respondents with answers 1-5 of table 1, and the extended sample additionally includes rows 6, 8, 9, and 13. *** p<0.001, ** p<0.01, * p<0.05.

**Table C. Summary statistics Emotional Stability**

|  | No | | | Yes | | | Difference | | |
| --- | --- | --- | --- | --- | --- | --- | --- | --- | --- |
|  | Original | Vignette baseline | Vignette extended | Original | Vignette baseline | Vignette extended | Original | Vignette baseline | Vignette extended |
|  |  |  |  |  |  |  |  |  |  |
| Female | 4.26 | 2.56 | 2.34 | 3.21 | 1.87 | 1.62 | 1.05*** | 0.69*** | 0.71*** |
|  |  |  |  |  |  |  |  |  |  |
| Age: 19 and older | 3.68 | 2.31 | 1.98 | 3.91 | 2.31 | 2.10 | -0.23 | -0.01 | -0.12 |
|  |  |  |  |  |  |  |  |  |  |
| Parents higher education | 3.84 | 2.32 | 2.10 | 3.80 | 2.32 | 2.03 | 0.04 | 0.00 | 0.07 |
|  |  |  |  |  |  |  |  |  |  |
| Nationality: |  |  |  |  |  |  |  |  |  |
| Dutch | 3.70 | 2.27 | 1.96 | 4.17 | 2.42 | 2.29 | -0.47** | -0.15 | -0.33* |
| Belgian | 3.91 | 2.34 | 2.08 | 3.27 | 2.10 | 1.81 | 0.64*** | 0.24 | 0.27 |
| German | 3.70 | 2.34 | 2.02 | 3.98 | 2.26 | 2.07 | -0.27 | 0.08 | -0.05 |
| Other nationality | 3.87 | 2.29 | 2.08 | 3.60 | 2.39 | 1.91 | 0.26 | -0.10 | 0.17 |
|  |  |  |  |  |  |  |  |  |  |
| Math Major in high school | 3.69 | 2.22 | 1.92 | 4.00 | 2.46 | 2.22 | -0.31* | -0.24 | -0.30* |
|  |  |  |  |  |  |  |  |  |  |
| Math entry test: 8-14 | 3.72 | 2.27 | 1.96 | 3.86 | 2.34 | 2.11 | -0.14 | -0.07 | -0.15 |
|  |  |  |  |  |  |  |  |  |  |

Note: “No” indicates the value 0 and “Yes” implies the value 1 for the variables displayed in the rows. The baseline sample contains only respondents with answers 1-5 of table 1, and the extended sample additionally includes rows 6, 8, 9, and 13. *** p<0.001, ** p<0.01, * p<0.05.

**Table D. Summary statistics Risk Preference**

|  | No | | | Yes | | | Difference | | |
| --- | --- | --- | --- | --- | --- | --- | --- | --- | --- |
|  | Original | Vignette baseline | Vignette extended | Original | Vignette baseline | Vignette extended | Original | Vignette baseline | Vignette extended |
|  |  |  |  |  |  |  |  |  |  |
| Female | 5.02 | 3.16 | 3.22 | 4.52 | 2.93 | 2.96 | 0.50*** | 0.23*** | 0.25*** |
|  |  |  |  |  |  |  |  |  |  |
| Age: 19 and older | 4.68 | 3.00 | 3.02 | 4.92 | 3.10 | 3.18 | -0.24* | -0.10 | -0.15* |
|  |  |  |  |  |  |  |  |  |  |
| Parents higher education | 4.76 | 3.06 | 3.11 | 4.81 | 3.05 | 3.10 | -0.06 | 0.01 | 0.01 |
|  |  |  |  |  |  |  |  |  |  |
| Nationality: |  |  |  |  |  |  |  |  |  |
| Dutch | 4.86 | 3.04 | 3.10 | 4.59 | 3.09 | 3.09 | 0.27 | -0.04 | 0.02 |
| Belgian | 4.79 | 3.06 | 3.10 | 4.87 | 3.00 | 3.08 | -0.08 | 0.06 | 0.02 |
| German | 4.90 | 3.09 | 3.13 | 4.61 | 2.98 | 3.04 | 0.30* | 0.11 | 0.09 |
| Other nationality | 4.66 | 3.02 | 3.06 | 5.20 | 3.16 | 3.21 | -0.54*** | -0.14 | -0.15 |
|  |  |  |  |  |  |  |  |  |  |
| Math Major in high school | 4.81 | 3.02 | 3.08 | 4.79 | 3.10 | 3.14 | 0.02 | -0.08 | -0.06 |
|  |  |  |  |  |  |  |  |  |  |
| Math entry test: 8-14 | 4.79 | 3.04 | 3.10 | 4.84 | 3.11 | 3.13 | -0.05 | -0.07 | -0.03 |
|  |  |  |  |  |  |  |  |  |  |

Note: “No” indicates the value 0 and “Yes” implies the value 1 for the variables displayed in the rows. The baseline sample contains only respondents with answers 1-5 of table 1, and the extended sample additionally includes rows 6, 8, 9, and 13. *** p<0.001, ** p<0.01, * p<0.05.
